# Control of Dental Caries in Children and Adolescents Using Fluoride: An Overview of Community-Level Fluoridation Methods

**DOI:** 10.3390/pediatric16020021

**Published:** 2024-03-27

**Authors:** América Patricia Pontigo-Loyola, Martha Mendoza-Rodriguez, Rubén de la Rosa-Santillana, Maria Gracia Rivera-Pacheco, Horacio Islas-Granillo, Juan Fernando Casanova-Rosado, María de Lourdes Márquez-Corona, José de Jesús Navarrete-Hernández, Carlo Eduardo Medina-Solís, David J. Manton

**Affiliations:** 1 Academic Area of Dentistry of Health Sciences Institute, Autonomous University of Hidalgo State, Pachuca 42160, Mexico; americap@uaeh.edu.mx (A.P.P.-L.); martha_mendoza2138@uaeh.edu.mx (M.M.-R.); rubenrs@uaeh.edu.mx (R.d.l.R.-S.); m.rivera.pacheco@umcg.nl (M.G.R.-P.); hislasg@uaeh.edu.mx (H.I.-G.); lmarquez@uaeh.edu.mx (M.d.L.M.-C.); 2 Department of Cariology, Centre for Dentistry and Oral Hygiene, University Medical Centre Groningen, University of Groningen, 9713 GZ Groningen, The Netherlands; d.j.manton@umcg.nl; 3 School of Dentistry, Autonomous University of Campeche, Campeche 24039, Mexico; jfcasano@uacam.mx; 4 Advanced Studies and Research Center in Dentistry “Dr. Keisaburo Miyata” of Faculty of Dentistry, Autonomous University of the State of Mexico, Toluca 50130, Mexico

**Keywords:** oral health, dental caries, fluoride, prevention, children, adolescents

## Abstract

The maintenance of oral health is a crucial aspect of general well-being; however, a significant proportion of the worldwide population experiences a range of oral diseases. Dental caries is a highly prevalent non-communicable disease globally, especially in children and adolescents. Fluoride is involved in the control of dental caries, primarily by decreasing the critical pH for dental hard tissue dissolution and decreasing enamel solubility. Due to the substantial data supporting the efficacy of fluoride in controlling dental caries, many community-level fluoridation initiatives have been devised and executed as global public health preventive interventions. These initiatives encompass the fluoridation of water, salt, and milk. Water fluoridation is considered safe and effective when fluoride levels are maintained within the recommended range (0.6 to 1.1 mg/L). Salt fluoridation has a cariostatic potential similar to that of water fluoridation, and a fluoride concentration of 250 micrograms per gram in salt is not associated with an increased risk of developing dental fluorosis. However, there is currently an effort to reduce the consumption of table salt in order to mitigate the harmful effects of excessive salt consumption. It has been hypothesized that fluoride food supplementation, such as fluoridated milk, is associated with a decrease in caries experience in permanent teeth; however, the effect is not clear in primary teeth. Public-level fluoride interventions are more cost-effective than the operative care of caries lesions and limit the burden of care. The administration of fluorides should be conducted using safe methods, limiting ingestion, and adhering to the guidelines set by international and national health agencies in each country. This is particularly important when considering children with developing dentitions. Fluoride is an important tool in the control of dental caries, but it is crucial to combine it with good oral hygiene, a healthy diet, and regular visits to a dental professional to maintain long-term oral health.

## 1. Introduction

Oral health is associated with a multitude of human existential dimensions, encompassing the capacity for verbal communication, displaying facial expressions, olfactory and gustatory sensations, tactile perception, mastication, deglutition, and the ability to transmit a range of emotions without experiencing discomfort, pain, or afflictions of the craniofacial complex [1]. The World Health Organization (WHO) defines oral health as the ‘absence of oral and facial discomfort, oral infections and lesions, periodontal diseases, dental caries, tooth loss, and other ailments that restrict an individual’s oral health, including their ability to bite, chew, smile, speak, and maintain psychosocial well-being’ [2]. The WHO’s recent 2022 publication, entitled “Global Report on Oral Health”, sheds light on the concerning global situation of oral health, posing a significant challenge for healthcare systems. The authors advocate for immediate actions to include oral health with other non-communicable diseases (NCD) and highlight the ‘human right to oral health’ and the necessity of achieving universal health coverage [3].

A significant proportion of the world’s population suffers from oral diseases throughout their lives, causing pain, discomfort, disfigurement, and even death. These issues are more prevalent in socially disadvantaged populations and are considered serious public health problems in many parts of the world, with much of the disease untreated [4,5,6]. Despite efforts aimed at improving oral health, nearly half of the world’s population experiences disability due to oral conditions (age-standardized prevalence: 48.0%) [7]. For example, untreated caries lesions in permanent teeth were the most prevalent condition in the Global Burden of Diseases Study in 2015 (age-standardized prevalence: 34.1%), affecting 2.5 billion people worldwide. Furthermore, the age-standardized prevalence of untreated caries lesions in primary teeth was 7.8% (573 million affected individuals) [7]. More recently (2017), it was estimated that 3.5 billion individuals had oral diseases globally (95% uncertainty interval [95% UI]: 3.2 to 3.7 billion), with 2.3 billion having untreated caries lesions in permanent teeth and 532 million children having untreated caries lesions in primary teeth [8]. Therefore, dental caries is one of the most common, yet often neglected, NCDs among children and adolescents worldwide, with a large proportion having high treatment needs [9].

Dental caries refers to both the disease process and the clinical outcome of the process—the caries lesion [10]. Dental caries is a behaviorally moderated diet-dependent microbial disease that requires a cariogenic biofilm and regular dietary exposure to fermentable carbohydrates—socio-demographic (disadvantage) factors also influence the disease significantly. Due to frequent consumption of free sugars, the oral biofilm becomes dysbiotic, and acidogenic, aciduric, and cariogenic microbes flourish, leading to increased caries risk. The dysbiotic cariogenic biofilm metabolizes fermentable carbohydrates, decreasing the pH and relative saturation with respect to the tooth mineral, leading to demineralization of the hard tissues. The net balance of demineralization and remineralization, when favoring demineralization, causes a net loss of the tooth mineral, resulting in a visible caries lesion (white spot lesion) [11,12].

Dental caries can be controlled largely through community and individual actions, and doing so has positive effects on well-being and long-term costs for individuals and national healthcare systems; however, the ongoing high prevalence of the disease indicates that this process is not simple [9]. Children with high caries experience have a high risk of ongoing risk into adulthood; therefore, it is crucial to intervene at an early age to maintain healthy behaviors and better oral health into adulthood [13,14]. Caries control refers to the various strategies and methods used to control and limit caries risk and lesion development [15]. There are several effective methods for caries control that aim to reduce the risk of caries by focusing on different aspects of the disease process, such as biofilm removal and microbial modification and the introduction of a healthy, low-sugar diet.

The aim of this paper is to describe the community-level methods using fluorides for the control of dental caries in children and adolescents. According to the PICO strategy, the following research question was defined: “(P) in children and adolescents, (I) what are the methods for primary prevention of dental caries using fluorides, (C) using community strategies (O) to improve and maintain dental health?”

## 2. Review

### 2.1. Use of Fluorides in Caries Control

Fluoride is a compound that decreases the solubility of dental enamel when incorporated into the hydroxyapatite crystal, thereby lowering its solubility and the critical pH for dissolution. Fluoride also exerts its anticariogenic action by being in solution and changing the saturation characteristics with respect to the tooth mineral in the biofilm fluid at the tooth surface and within the tooth mineral by enhancing remineralization and decreasing demineralization and, when at a sufficient concentration, by inhibiting bacterial metabolism of carbohydrates [16,17]. The possible mechanisms that are believed to explain the cariostatic effect of fluoride include the ability to suppress the generation of acid by bacteria, the ability to inhibit the development of extracellular polysaccharides (EPS), the ability to prevent the demineralization of enamel, and the ability to stimulate remineralization. These mechanisms may function individually or in combination. An experiment showed that the application of NaF totally stopped the process of demineralization without having any impact on the composition and growth of the biofilm. The reported decrease in acid production or EPS volume, or a shift in the de/remineralization balance, may be responsible for this protective impact [18]. The role of fluoride in caries control is well established, and adequate and regular fluoride exposure is beneficial for maintaining dental health [19].

Fluoride can be delivered either systemically or topically to prevent dental caries, with the most important action being topical. Toothpastes, mouth-rinses, and a variety of professionally administered products are all examples of ways that fluoride can be delivered topically. When it comes to administering fluoride through systemic methods, such as reticulated water fluoridation, they result in the fluoride being ingested and remaining in the saliva, as well as a small amount being incorporated into the tooth mineral during amelogenesis. However, the main impact is still at the surface of the tooth. In addition to the main topical cariostatic effect of fluorides, regular use of fluoride toothpaste enhances the effect, while excessive fluoride intake during tooth formation can increase the risk of dental fluorosis, a systemic effect on the ameloblasts of developing teeth leading to hypomineralized enamel lesions [20,21,22,23].

### 2.2. Fluoride Delivery Vehicles

It is nearly impossible to determine what is the topical and what is the systemic effect of fluoride, but ingested fluoride only has a minor benefit. As a result of this health benefit and the concomitant topical effect, numerous community-level fluoridation programs have been developed and implemented as a public health preventive measure worldwide, including water, salt, and milk fluoridation. These programs have been successfully evaluated and have reduced the need for curative and restorative treatment-based programs, especially in areas with inadequate access to oral care [23,24].

#### 2.2.1. Water Fluoridation

Water fluoridation is a public health intervention dating back to the mid-20th century and is considered one of the top 10 public health achievements [25,26]. Historically, scientists noticed that residents of areas with naturally fluoridated water had fewer caries lesions. One of the early studies on water fluoridation was conducted in Colorado Springs, United States, in the 1900s. Dentist Frederick S. McKay and chemist Henry V. Churchill researched a dental condition called “Colorado brown stain”, which turned out to be the visual effect of dental fluorosis. Throughout the 1920s and 1930s, researchers began to seek the optimal fluoride dose to prevent caries without causing dental fluorosis. The work of researchers such as H. Trendley Dean helped establish the relationship between the fluoride concentration in water and the prevalence of dental caries. Studies in the early 1940s demonstrated that adding fluoride to drinking water at optimal concentrations significantly reduced the prevalence of dental caries without causing severe dental fluorosis. This led to the initiation of water fluoridation programs in various communities in the United States, with Grand Rapids, Michigan, being the first city to start a water fluoridation program in 1945 [27,28,29,30]. Other significant programs followed in other parts of the world, for example, in the United States, in Newburgh in 1945 and in Evanston, Illinois, in 1946; in Canada in Brantford, Ontario in 1945; in the Netherlands in 1953; in New Zealand in 1954 [31]; in the United Kingdom, the first substantial introduction was in Birmingham and a few other cities in 1964 [32]; and in the German Democratic Republic in 1959. Subsequently, extensive water fluoridation programs have been introduced in Australia, Brazil, Chile, Colombia, Canada, the Hong Kong Special Administrative Region of China, Ireland, Israel, Malaysia, New Zealand, Singapore, the United Kingdom, and other locations. More recently, new programs have been introduced in large urban areas in the southern and western United States, including Los Angeles (in 1999), Las Vegas (in 2000), Sacramento (in 2000), and San Antonio (in 2002) [31]. However, water fluoridation is largely absent in European countries.

Water fluoridation is considered safe and effective when fluoride concentrations are maintained within the recommended range. The World Health Organization recommends a maximum fluoride level in drinking water of 1.5 mg/L. It is indicated that to establish national standards for water fluoridation, it is crucial to consider climatic conditions, water consumption, and fluoride ingestion via other sources, such as food and the environment. Obtaining the reference value can be challenging in some circumstances despite available treatment technology in areas with high natural fluoride levels [33]. The optimal fluoride level in drinking water varies for some countries; in Australia, the current guidelines from the National Health and Medical Research Council recommend water supply fluoridation within the range of from 0.6 to 1.1 mg/L, depending on the area’s average temperature [27]. In the United States, the U.S. Public Health Service currently recommends an optimal fluoride concentration of 0.7 mg/L to maintain the benefits of caries prevention while reducing the risk of dental fluorosis [34]. This has been shown to be effective in reducing the incidence of caries amongst children and adolescents by 20 to 40% [35], or even by up to 60% [27]. Due to its universal reach and passive absorption mechanism, water fluoridation has the potential for equitable impact, benefiting everyone, especially those with poorer dental health and/or less access to other health promotion and prevention avenues [28,29].

In a Cochrane systematic review on the relationship between water fluoridation and dental caries, which included 107 studies, the authors found that the results from the severity of caries data indicated that the initiation of water fluoridation led to reductions in caries experience on primary dentition by 1.81 for the decayed, missing, or filled teeth (dmft) index and 1.16 for the Decayed, Missing, or Filled surfaces (DMF) index on permanent dentition. Compared to the mean values of the control group median, this resulted in a 35% decrease in the DMFT index and a 26% decrease in the DMFS index. The percentage of children with dmft = 0 increased by 15% in the primary dentition and by 14% in the permanent dentition (DMFT = 0). However, there is insufficient information to determine whether the initiation of a water fluoridation program leads to changes in caries inequalities across different socioeconomic levels or if the cessation of water fluoridation programs has an effect on caries levels [36]. In Australia, in a more recent systematic review, it was reported that water fluoridation had reduced dental caries by 26% to 44% in children, adolescents, and adults, benefiting everyone regardless of age, income, or access to dental care [27].

Overall, water fluoridation is used in many developed countries where large urban populations have access to treated and reticulated water supplies [37]. One of the most cost-effective public health interventions for reducing dental caries is water fluoridation. Studies show that water fluoridation is cheaper than dental caries treatment of the disease increment it prevents. Water fluoridation improves oral health and reduces the need for costly dental procedures, including restorations, root canal treatments, and extractions. In Australia, water fluoridation saves between AUD 7 and 18 in dental treatment expenses for every dollar spent [27]. It has recently been estimated that for every dollar invested in water fluoridation by the government, the estimated long-term economic value of health service savings ranges from AUD 1.1 (population ≤ 300) to AUD 16 (population ≥ 2000) due to reductions in the need for dental caries treatment and associated hospitalizations [38]. In the United States, the savings from community water fluoridation programs associated with prevented caries due to fluoridation are estimated to be USD 32.19 per capita [39]. In the United States, where 60.5% of the total population has access to fluoridated water, the annual per capita cost was estimated at USD 0.50; ranging from USD 2.94 in small communities (<5000) to USD 0.46 in larger communities (>20,000) [40]. However, water fluoridation is not feasible in countries without a centralized water supply.

#### 2.2.2. Salt Fluoridation

In 1955, Switzerland became the first country in the world to begin fluoridating table salt (NaCl) in order to minimize the incidence of dental caries. This decision was made in the context of the country’s prior experience with iodized salt, which was found to be effective in preventing goiter. The acceptance of fluoridating salt was made easier as a result of the following factors: (1) the availability of scientific evidence from successful community trials; (2) recommendations regarding the topic made by the WHO and the World Dental Federation; (3) approval from the European Union for sodium and potassium fluoride to be used as food additives; and (4) adaptation to the local political, technological, and cultural environments [41]. The fluoridation of salt refers to the practice of adding fluoride to salt intended for human consumption. This practice has been legal in the United States since 1980–1982. The fluoridation of salt is carried out with the intention of preventing dental caries, particularly among lower socioeconomic groups. Fluoridated salt is distributed to consumers through a variety of ways, such as through household salt, school meals, commercial kitchens, and bread, and it has effects that are both topical and systemic. This technique is by far the most cost-effective strategy to prevent dental caries, and it has the potential to help billions of people worldwide [41,42].

Salt fluoridation has a cariostatic potential similar to that of water fluoridation (reducing caries prevalence by up to 50%). In Europe, significant proportions of the population have been reached only in Germany (67%) and Switzerland (85%). In Latin America, there are over 100 million users, and several countries have achieved a coverage from 90 to 99% [42]. An analysis of the evidence on the efficiency of salt fluoridation in controlling dental caries and the hazards of dental fluorosis found that salt fluoridation is successful in reducing caries prevalence [43]. If the fluoride concentration in salt is less than 250 micrograms per gram, there is no increased risk of dental fluorosis. However, any time that fluoride supplementation, fluoride-rich mineral waters, and fluoridated table salt are accessible at the same time, explicit advice on systemic fluoride supplementation should be offered to limit the risk of fluorosis [44].

A decrease in the prevalence of caries occurrence has been reported in several studies [45], including meta-analyses [46]. A literature review, based on 22 references, provided information on the preventive effects of salt fluoridation programs in Europe (Hungary, Switzerland, France, Germany) and in North, South, and Central America (Mexico, Colombia, and Jamaica). Most of the data were obtained from descriptive or retrospective epidemiological studies. The results indicated that in the absence of other methods of topical fluoride exposure, salt fluoridation led to a significant reduction in caries experience among treated children compared to a control group. The additional effect of salt fluoridation is reduced when fluoridated toothpastes are used frequently. It is reported that fluoridated salt does not significantly increase the prevalence of fluorosis, however, the side-effects are not always evaluated [47].

In countries such as the United States, after 70 years of water fluoridation, there are still communities that do not enjoy the benefits of water fluoridation. Therefore, salt fluoridation can be a complementary method to improve child dental health in areas where water fluoridation is not available. It is used worldwide and is recommended by the WHO when water fluoridation is not feasible, as it is equally effective in preventing and controlling dental caries [48]. Overall, a more realistic and equitable option for delivering fluoride to children in developing countries is the implementation of salt fluoridation, as it is relatively easy to implement, can complement salt iodization, and involves a relatively small increase in production costs. Additionally, salt has the important advantage of being consumed by the entire population and requiring a relatively simple distribution network [37]. The cost of salt fluoridation is estimated to be very low, ranging from EUR 0.02 to 0.05 per year per capita [42]. Other estimates mention that the costs of salt fluoridation would range from USD 0.023 to 0.038 per year per capita [40]. Salt fluoridation may be a complementary method to improve children’s dental health in areas where water fluoridation is not available. However, it is crucial to consider this within the current context that recommends reducing salt consumption. It is important to find a balance between the benefits of salt fluoridation for dental health and the need to control salt intake in general, taking into account public health recommendations. Additionally, concerns about excessive fluoride ingestion and its potential adverse health effects must be appropriately addressed. Therefore, any salt fluoridation program must be implemented with caution and vigilance, ensuring constant monitoring of fluoride levels and appropriate education on its use.

#### 2.2.3. Fluoride Supplements

After determining whether or not a child is at risk for dental caries, medical practitioners may advise parents to give their children fluoride supplements in locations where the natural fluoride concentration of the drinking water is insufficient to offer effective protection [49]. Before prescribing a fluoride supplement, it is necessary to investigate all possible sources of fluoride [50]. The American Dental Association (ADA) supports the daily use of fluoride supplements by children up to 16 years of age. These supplements typically come in the form of drops or (chewable) tablets, and they often need to be recommended by a dentist or pediatrician [51].

It has been suggested that the use of fluoride supplements is associated with a reduction in the incidence of caries in the permanent dentition compared to no fluoride supplementation. However, the impact that fluoride supplements have on the primary dentition is not completely understood. When compared to the use of topical fluorides, there is no discernible difference in the observed impact [52]. The use of systemic fluoride supplements is only beneficial if they are administered on a consistent basis and in accordance with established protocols in the years of development of the first 28 permanent teeth, which is normally between the ages of two and eight years. After this period of time, there is no longer any effect of systemic fluoride ingestion on the dentition [53]. On the other hand, there is no evidence to suggest that fluoride supplements that are taken by pregnant women are useful in preventing dental caries in their children [54].

It is not recommended to give fluoride supplements to infants who are younger than six months of age, as stated by the American Academy of Pediatrics [55]. After this age, newborns who are either breastfed or given formula should take a fluoride supplement that is appropriate for them. If the available drinking water in the area has a fluoride concentration lower than 0.3 parts per million (ppm), then infants would benefit from taking a fluoride supplement if at increased caries risk. This suggestion is based on limited scientific evidence, although it has been commonly believed for decades that the “optimal” fluoride consumption is between 0.05 and 0.07 milligrams of fluoride per kilogram of body weight (F mg/kg body weight). However, the scientific evidence supporting this guideline is weak. According to the findings of a long-term study, the estimated average daily fluoride intake for children without a history of caries and without fluorosis at the age of 9 was 0.05 mg F/kg body weight or less for the majority of the time during the first 48 months of life, and this level fell thereafter. In addition, fluorosis was not present in any of the children in the study [56].

There is little and contradictory evidence to support the claim that oral fluoride supplements can effectively prevent dental caries [57], with only a few studies investigating their preventive efficacy [58], and the effectiveness of various fluoride supplement alternatives, such as fluoridating milk, is still debatable. At the beginning of the 1950s, researchers in Switzerland, the USA, and Japan [59] began investigating the possibility of using milk as a carrier for fluoride. This idea had been circulating since the early 1950s. A further piece of evidence that supports this intervention is the post-eruptive effect of fluoride. Research conducted in vitro, in situ, and in vivo has indicated that the mechanism of action of fluoride may be primarily attributable to the fact that it has an influence on the kinetics of demineralization and remineralization of hard dental tissues [58,60].

According to a Cochrane review, there is low-quality evidence indicating that fluoridated milk may help prevent caries in the primary teeth of schoolchildren. Regarding the efficacy of caries experience reduction, just one study with substantial methodological limitations has been carried out. In addition, there was a lack of information regarding the possible adverse effects that could have been caused by the intervention. Before conclusive findings can be reached on the advantages of fluoridating milk, more controlled clinical research of a high quality is required [61].

Currently, children in kindergartens and schools in around 15 countries are receiving fluoridated milk through a variety of distribution methods as part of ongoing milk fluoridation initiatives that are now being carried out with the cooperation of the WHO and the Food and Agriculture Organization. The fluoridation of milk has been demonstrated (although with a low quality of evidence [61]) to be effective in controlling dental caries on a global scale. It has been advised that infants incorporate fluoride-enriched milk into their diet from an early stage, ideally before the age of 4 years and before the eruption of the first permanent molars [41]. This would protect and decrease the incidence of caries in primary teeth and would begin the process of protecting and reducing the incidence of caries in permanent teeth. Fluoridation milk programs have a relatively low cost, between EUR 2 and 3 per child per year. When the fluoride content in drinking water is suboptimal, children’s caries experience increases, and when a school milk program exists, milk fluoridation can be recommended as a preventive intervention for controlling caries [62].

This review sheds light on the unequal research on the systemic aspects of fluoridation, as a search (8 November 2023) in available Pubmed/Medline article titles using the keywords “fluoridated water”, “fluoridated salt”, and “fluoridated milk” yielded 800, 74, and 25 results, respectively, indicating more research on one topic compared to others, with certain regions of the world taking a more proactive role in this research domain. Furthermore, for example, the most recent article on fluoridated salt is from 2019, while for fluoridated water and milk, the articles are from 2023 and 2022, respectively. What have been consistent in these studies are the positive effects of fluoridation in controlling dental caries. It is vital to promote further research efforts that encompass both the positive and negative impacts of fluoridation.

## 3. Fluorosis Risk from Systemic Fluoride

Fluorosis is a condition that occurs due to excessive fluoride ingestion, leading to changes in the enamel of teeth and, in severe cases, affecting the skeletal system and other body functions [63]. The risk of developing fluorotic lesions of enamel due to the ingestion of recommended levels of fluoride during amelogenesis has been deemed acceptable when compared to the anticaries benefits of fluoride [53,64]. However, excessive fluoride intake can have negative effects on thyroid function, the skeletal system, the reproductive system, and cardiovascular health [65,66,67]. The risk of fluorosis depends on the dose, duration, and timing of fluoride exposure during tooth enamel formation [53,68]. Factors such as the amount of fluoride ingested according to the child’s weight and the duration of fluoride exposure during tooth development are important considerations [69,70]. It is worth noting that fluorosis is a dental disturbance, and the duration of fluoride exposure during tooth development is more significant than sporadic dose peaks [53,71].

Fluorosis can occur in areas with fluoridated water as well as in areas without it. Other sources of excessive fluoride intake include groundwater naturally rich in fluoride, the use of high-fluoride water in food preparation or irrigation of crops, and the consumption of products containing added fluoride [72,73,74]. It is important to note that the level of fluoride in public drinking water has been adjusted to minimize fluorosis and significantly decrease the incidence of dental caries at a relatively low cost [70,75]. The benefits of water fluoridation in preventing dental caries have been well-established, and the risks of dental fluorosis have been considered acceptable when contrasted with the anticaries benefits of fluoride [76]. In brief, while the risk of dental fluorosis exists with fluoride ingestion, it is important to consider the benefits of fluoride in controlling dental caries. The risk of fluorosis depends on factors such as the dose, duration, and timing of fluoride exposure during tooth development. Excessive fluoride intake can have negative effects on various body functions. It is essential to balance the benefits and risks of fluoride ingestion and implement appropriate measures to minimize the risk of fluorosis.

## 4. Conclusions

It is important to note that the effectiveness of caries prevention methods using fluorides added to water or foods may vary depending on individual factors, and it is important to use appropriate quantities recommended by international and national health agencies as well as by healthcare professionals, especially in children. Oral health professionals can provide guidance on effectively incorporating fluoride into a child’s oral care routine. Excessive fluoride ingestion can compromise dental health and also lead to other general health problems. When prescribing dietary fluoride supplements to children, to minimize the risk of dental fluorosis, caution should be exercised to avoid excessive fluoride ingestion relevant to the child’s age and tooth development stages. This is especially important if there are other relevant sources of fluoride intake such as drinking water, salt, milk, and the use of personal care products. Fluoride is an essential weapon in the war against caries, but in order to keep one’s teeth in good condition over their life course, it is essential to combine fluoride use with proper oral hygiene practices, a healthy low-sugar diet, and regular dental visits.
